# Determinants of binge drinking in a permissive environment: focus group interviews with Dutch adolescents and parents

**DOI:** 10.1186/1471-2458-13-882

**Published:** 2013-09-24

**Authors:** Astrid Jander, Liesbeth Mercken, Rik Crutzen, Hein de Vries

**Affiliations:** 1 Maastricht University, School of Public Health and Primary Care CAPHRI, Maastricht, The Netherlands

**Keywords:** Alcohol, Adolescents, Binge drinking, Focus group interviews, Parents

## Abstract

**Background:**

Compared to other European countries, the Netherlands score among the highest of binge drinking rates of 16 to 18 year old adolescents. Dutch adolescents aged 16 are legally allowed to buy and consume low strength alcoholic beverages. This study focused on determinants of binge drinking in such a permissive environment from the perspectives of adolescents and parents.

**Methods:**

Focus group interviews were conducted with adolescents aged 16 to 18 (N = 83), and parents of adolescents from this age group (N = 24). Data was analysed using thematic analyses methods.

**Results:**

Most reasons adolescents mentioned for drinking were to relax, increase a good mood and to be social. Also peers around them influenced and increased adolescents’ drinking. Comparing adolescents and parental statements about their perspectives how alcohol use is handled and accepted by the parents we found that generally, those perspectives match. Parents as well as adolescents stated that alcohol use is accepted by parents. However, when looking at essential details, like the acceptable amounts that children may consume, the perspectives differ enormously. Adolescents think their parents accept any amount of drinking as long as they do not get drunk, whereas parents reported acceptable limits of 1 or 2 glasses every two weeks. Parents further indicated that they felt unsupported by the Dutch policies and regulations of alcohol use. Most of them were in favour of an increase of the legal purchasing age to 18 years.

**Conclusions:**

Parents and adolescents should both be targeted in interventions to reduce alcohol use among adolescents. In particular, communication between parents and children should be improved, in order to avoid misconceptions about acceptable alcohol use. Further, adolescents should be supported to handle difficult social situations with peers where they feel obliged to drink. Additionally, revisions of policies towards a less permissive standpoint are advised to support parents and to impede availability of alcoholic beverages for adolescents/children younger than 18 years.

## Background

Binge drinking (i.e., 4/5 or more standard glasses of alcohol for women/men at one occasion) is a growing problem in Europe. A survey showed 24% of all 15–24 year old Europeans reported binge drinking at least once a week [1]. In the Netherlands the frequency of adolescent binge drinking is among the highest in Europe [2,3]. A recent study showed that 59% of all 16 year old and 71% of all 17–18 year old Dutch adolescents have had at least one binge drinking occasion in the past 30 days [4]. The Netherlands is one of few countries in the world that have a legal purchasing age of 16 years for low strength alcoholic beverages like beer and wine [5]. This implies that regulations concerning alcohol purchases in this underage group are absent and that it is the responsibility of parents and adolescents to regulate their alcohol consumptions. In this respect, Dutch adolescents and parents face a unique situation, in which families have to deal with the alcohol use of adolescents in a permissive environment, where underage adolescents are legally allowed to buy alcohol and it is also accepted that adolescents drink at a relatively young age. These adolescents often still live at home in contrast to, for example, the US where the legal drinking age is much higher and more adolescents already live on their own when they enter the legal drinking age. Not much research has focused on this specific target group yet, so adequate knowledge about determinants of alcohol use is lacking. It is important to investigate in more detail what determines alcohol consumption and, more interestingly, binge drinking in this specific group and what the role of parents is, in order to give recommendations for possible interventions to reduce alcohol use in these adolescents.

It is important to reduce alcohol use in adolescents, because it is associated with a variety of negative consequences, like, getting into fights, experiencing dating violence, having forced intercourse, having considered or attempted suicide, and using other (illicit) drugs [6,7]. In addition, binge drinking also negatively effects school performance [6], impairs learning and memory, and can result in permanent brain damage and cognitive deficits [8]. In order to fight these consequences we need to know what the determinants of alcohol use in this age group are.

Studies and reviews about determinants of alcohol use and binge drinking during adolescence and young adulthood identified several factors that influence alcohol consumption. Firstly, several studies addressed personal factors. One study examined the predictive value of constructs of the theory of planned behavior [9] in fifth to eighth grade students and found intention to drink alcohol accounted for 26% of the variance in alcohol use, while attitudes, subjective norms and perceived control explained 76% of the variance of intention to drink [10]. Another study using an extended version of the theory of planned behavior showed that in undergraduate students attitudes and anticipated regret were strong predictors explaining 58% of variance in intention not to binge drink and that past behavior significantly predicted actual binge drinking behavior, explaining 32% of the variance [11]. A more recent study suggests that beliefs of undergraduate students predicted intentions to binge drink in the evening and actual drinking behavior [12]. Those beliefs were that friends approve binge drinking, lack of money would make it difficult to binge drink, getting drunk is enjoyable, sports teams approved binge drinking, and that celebrating, drinking patterns, and environment make it easier to binge drink. These beliefs strongly overlap with so called drinking motives based on the motivational model of alcohol use [13]. In a review about drinking motives of 10 to 25 year old adolescents, the social drinking motive, which is drinking together with other people in order to get socially rewarded, was found to be related to moderate alcohol use. Enhancement drinking motives, which is drinking to enhance a positive mood, were related with heavy drinking, and coping motives, which means drinking to deal with negative emotions, with alcohol-related problems [14]. Finally, adolescents who score high on a sensation seeking and impulsivity scale also tend to engage in problem drinking more often than adolescents that score low [15]. An increase in sensation seeking and risk-taking propensity was predictive of greater odds of alcohol use [16]. It is interesting to find out what personal factors play a role in 16 to 18 year old adolescents that are allowed to drink alcohol to see whether the factors are comparable or perhaps that additional factors play a role.

Secondly, studies examining peer influences on binge drinking revealed that the presence of friends increased the likelihood that a certain event would become a heavy drinking event by 2.4 times [17]. Perceived friends’ drinking behavior and the friends’ normative standards were the strongest predictor of alcohol use for female adolescents [18]. There is some evidence that not just peer influence leads to similar drinking patterns in adolescents, but also peer selection [19], which is selecting similar others as friends. However, the results concerning peer selection are mixed: in young adolescents (13 to 14 years) peer influences play a dominant role, but with increasing age peer selection becomes more important [20]. Other studies have found that both processes are important and stable over time [21], but also that peer influences are more essential than peer selection [22]. Furthermore, there is evidence that the influence of drinking friends on regular alcohol consumption of adolescents increases when adolescents grow older [23]. Assuming that peers do have a big influence on adolescent drinking, it is interesting to know how Dutch adolescents perceive this influence.

Finally, parental influences have been subject of study in order to explain binge drinking. One study found that adolescents with substance using peers were at greater risk of using alcohol only when their parents reported problems with alcohol. When parents did not have problems with alcohol use, adolescents with substance using peers were less at risk of using alcohol [24]. Furthermore, several other studies emphasize that certain parenting practices (i.e., specific and goal directed behaviors parents perform to socialize their children) positively influence alcohol consumption of adolescents. Parental monitoring, whether or not parents control and monitor the activities and whereabouts of their offspring, and parental disapproval of heavy drinking was associated with less heavy alcohol consumption in adolescents [25]. Similarly, having strict rules concerning alcohol consumption seems to prevent adolescents from starting heavy alcohol consumption [26]. Stricter alcohol rules are associated with less alcohol consumption and binge drinking in adolescents [27]. Studies about communication between parents and children about alcohol intake have shown beneficial effects on alcohol consumption [28] as well as non-effective or even detrimental effects [29]. A systematic review [30] of parenting factors associated with reduced adolescent alcohol use further found that parental modeling, limiting the alcohol availability to the child, the parent–child relationship quality, parental involvement and general communication were associated with delayed alcohol initiation and reduced levels of later drinking by adolescents.

However, these reviews and studies have only focused on younger adolescents aged 9 to 16 years [10,16,24,26,31] or older than 18 years [15,17,32], or adolescents with a broad range of age (10 to 25 years) [14]. Also, these studies used a variety of alcohol measures that often lack a clear definition, like heavy alcohol use, problem drinking, excessive use or heavy episodic drinking. To our knowledge, there has not been any research on determinants of binge drinking in the age group from 16 to 18 years in countries with a legal drinking or purchasing age of 16, such as the Netherlands. We therefore chose to conduct focus group interviews with the target group. Focus group interviews allow detecting information when little is known about a certain topic in a specific target group [33]. Additionally, influences of parents for this particular age group are also less explored. One may argue that their influence is declining for this age group [23], yet, since most of the 16–18 year olds are still living with their parents [34] it is relevant to know whether parents still perceive a parenting role concerning binge drinking.

In this paper, we combined focus group interviews conducted with 16 to 18 year old adolescents with focus group interviews with parents of adolescents from this age group. This allowed us to get a broad and detailed picture what determinants of alcohol use in this age group might be relevant and how alcohol use is managed in Dutch families. Further, we investigated differences and similarities in viewpoints of adolescents and parents, to provide useful insight into possible leads for further research and interventions.

## Methods

### Design

Nine focus group interviews with adolescents were conducted in schools within groups of 6 to 13 people. To stimulate group discussion about alcohol use and binge drinking we posed open-ended questions. Two researchers were present in all focus group interviews. One served as a discussion leader who guided the discussion until all questions were exhaustively answered. The second took notes and checked whether all questions topics were covered.

We held two focus group interviews with parents (twelve and six parents, respectively) using the same design as used in the interviews with the adolescents. Furthermore, we conducted eight one-on-one interviews (either on the telephone or face-to-face) asking the same questions as in the focus group interviews.

#### ***Recruitment and participants***

Fifteen schools of secondary vocational and pre-university education (adolescents in these schools were aged respectively 16 to 20 years and 13 to 18 years) were asked to participate in our study in order to get a representative sample of Dutch adolescents. In total, five schools took part in this study (response rate: 33%), located in four regions of the Netherlands (east, south, west and middle). Adolescents were recruited through a teacher and were told beforehand that the focus group was about alcohol consumption. As pupils who were in the same classes participated in the focus groups, two adolescents were still 15 years old, eight were 19 and three already 20 years old. However, the vast majority of students (N = 60) were within the age range of 16 to 18 years old. Every adolescent who took part in a focus group received a letter to give to their parents, in which the parents were invited to take part in a focus group interview. Four parents indicated interest after receiving the letter from their child (response rate: 5%). More parents were recruited with the help of an advertisement in the local newspaper (2 responses), an advertisement on the notice board in the academic hospital (2 responses), via the parents’ council (12 respondents) and the first aid association (6 respondents). All parents had children in the age group 16 to 18 years. If parents also had children outside this age group we indicated that they should be talking about their 16 to 18 year old children during the interview. Because some parents were not able to attend the focus group interviews, we decided to hold one-on-one interviews with these parents. We followed the same procedure as held during the focus group interviews, except that only discussions were possible between the interviewer and the participant and not amongst participants as in the focus group interviews.

#### ***Procedure***

Before the focus group started, adolescents were informed that the interviews would be recorded on tape, which would only be used for research purposes and not be accessible to anyone outside the research team. It was stressed that the opinion of the adolescents was important and that there were no right or wrong answers to the questions. Participants started by filling out a short questionnaire to assess some demographic variables (i.e. gender, educational level, age). Dependent on the answer to the question if they had engaged in binge drinking in the last month (“How often did you have 4 or more (women) or 5 or more (men) glasses of alcohol at one occasion in the previous 30 days?” 1 = “not at all” to 5 = “more often than 6 times”) we divided them into one of two groups. Adolescents got a blue or a yellow card that they had to put down in front of them so the interviewer could see which group each adolescent belonged to. The “blue group” had at least one binge drinking occasion and the “yellow group” had no binge drinking occasion in the previous 30 days. Both groups stayed in one focus group. This division enabled us to ask specific questions to only those adolescents who indicated engaging in binge drinking and other specific questions to only those who did not engage in binge drinking. Furthermore, it was possible to stimulate discussion between the groups on certain aspects one of the groups mentioned. This procedure was used to identify factors that lead to binge drinking and factors that prevent binge drinking in adolescents. After the participants had been divided into one of the two groups, the group interview started with posing the open questions to the group. When all questions had exhaustively been answered, the participants were thanked and received a voucher worth €7,50.

The parents also received a short questionnaire before the group interview started. The questionnaire was slightly adapted, asking for their gender, educational level, number of children, and the age of the children. Parents were also informed that the interview would be tape recorded. After filling in the questionnaire, the focus group started with posing open questions to the group. When all questions were answered, the parents were thanked for participation and received a voucher of €20.

#### ***Focus group interviews***

The interviews were semi-structured. The main topics of the interviews with the adolescents are summarized in Table 1, the main topics of the interviews with the parents in Table 2. Questions were written down in advance to facilitate the interviewers and to allow for similarity in main questions asked. The interviews, however, were held in a very open manner to ensure the natural flow of the discussion.

**Table 1:** Interview schedule adolescents (with predefined themes)

**Theme**	**Example questions**
Attitude	*What do you think of binge drinking*? *Are there any benefits*? *Are there any disadvantages*?
Social influences	*Which people like*/*approve that you are binge drinking*?
	*Do these people binge drink themselves*? *If yes*, *when*? *What happens then*?
	*Which people dislike*/*disapprove that you are binge drinking*? *How do they let you know*? *Do these people binge drink*?
Social network	*Are there differences in opinion or influences of your friends on your binge drinking behavior*?
	*Friends in school or outside school*
	*Older*/*younger friends*
	*Boyfriend*/*girlfriend*
	*Siblings*
First time binge drinking	*When and why was the first time you have been binge drinking*?
	*How was this*? *Who was involved*? *Did that had any consequences* (*punishment of parents*, *approval of friends*)?
Self efficacy	*When is it easy*/*hard not to binge drink*?
Action plans	*What do you do when you do not want to binge drink*? *How do you handle difficult situations*?
Environmental factors	*When*/*in which situations do you usually binge drink*?
	*When*/*in which situations do you drink alcohol without binge drinking*?
	*What causes you to drink*? *What withholds you from drinking*?
Attitude parents	*What do your parents think about you binge drinking*?
Monitoring parents	*Do your parents know where and with whom you are*? *Do you talk about e*.*g*. *when you have to be home in the night*?
Rules parents	*Are you allowed to drink alcohol at home*? *Are there any rules concerning your alcohol consumption in general*?
Communication	*Do you communicate with your parents about alcohol* (*how much you can drink*)?
Behavior parents	*Do your parents drink alcohol*? *If yes*, *when*/*how much*? *Do your parents provide you with alcohol*?

**Table 2:** Interview schedule parents (with predefined themes)

**Theme**	**Possible questions**
Attitude towards adolescent alcohol consumption	*What is your opinion about alcohol use in adolescents*?
	*Are there any pros of alcohol consumption*? *Are there any cons*?
Attitude towards adolescent binge drinking	*What is your opinion about binge drinking in adolescent*?
Knowledge about risks /consequences	*What do you know about the consequences of binge drinking*, *both long term and short term*?
Risk perception	*How serious do you think these consequences are*?
Rules	*Do you handle certain rules concerning the amount of alcohol that your child may drink*?
	*Do you handle any rules concerning the times that your child has to be home when it goes out*?
Beliefs about effective prevention of binge drinking	*What do you think is a good way do decrease binge drinking in your child*?
	*Do you think it is necessary to decrease binge drinking in your child*?
Beliefs about own influence	*Do you think that you still have influence on your child*’*s alcohol consumption*?
Actions	*What exactly do you do to decrease binge drinking in your child*?
Consequences	*What do you do if you realize your child has been binge drinking*? *Are there any consequences*?
Needs	*Do you sometimes think you could need some help with the alcohol education of your child*?
	*How should that help look like*?

#### ***Data analysis***

The audio taped interviews were transcribed and analyzed using QSR NVivo 8 software for qualitative data (http://www.qsrinternational.com). The aim of this study was not to test hypotheses or a theory but to obtain insight into determinants of adolescent binge drinking, using both the adolescent and the parent perspective. Consequently, we used a data driven thematic approach [35]. Two researchers read the transcripts repeatedly in order to get familiar with the data. Using the QSR NVivo 8 software, transcripts of all interviews were coded into themes. After all transcripts were coded once, the transcripts were checked again against the codes. Text parts could be coded under more than one theme. Codes where then, where possible, grouped together to form main themes and sub themes (Tables 3 and 4). Some discussion themes were predefined in the interview schedule (Tables 1 and 2).

**Table 3:** Final main and sub themes adolescents (predefined themes + themes that emerged from the data)

**Main themes**	**Sub themes**
Reasons not to binge drink	Negative experiences
	Environment
	Sports
	Expectancies
Influence of other people on non-binge drinking behavior	Social influence beliefs
	Environment
Reasons to start and continue drinking	Motives
	Environment
	Expectancies
Influence of other people on drinking behavior	Social influence beliefs
	Social network
Difficulties when trying not to binge drink	Cues
	Barriers
Parental attitude towards alcohol consumption	Parental attitude
	Drinking behavior parents
Influence of parents on alcohol consumption	Alcohol socialization
	First drink
	Awareness parents
	Drinking at home
Rules concerning alcohol consumption	Rules

**Table 4:** Final main and sub themes parents (predefined themes + themes that emerged from the data)

**Main themes**	**Sub themes**
Attitude towards alcohol	Pros
	Cons
	Own responsibility
Attitude towards binge drinking	Attitude towards binge drinking
Knowledge about drinking	Consequences (risks)
Parenting practices	Communication
	Rules
	Availability at home
	Consequences
Bringing down alcohol intake	Own drinking
	Communication
	Rewarding
	Schools
Responsibility	National campaigns
	Availability in environment
	policy

#### ***Ethics approval***

Ethical approval of the Regional Medical Ethics committee in the Netherlands was not necessary, because participants in this study were not “subjected to procedures or required to follow certain rules of behavior” (http://www.ccmo-online.nl/main.asp?pid=43&thid=57&catid=2).

## Results

### Interview adolescents

#### ***Questionnaire***

Nine interviews were held with 47 adolescents from secondary vocational education (three schools) and 36 adolescents with a pre-university educational background (two schools). The study sample comprised of 50 boys and 33 girls (mean age 17.2 years). The majority identified themselves as binge drinkers (65%, n = 54). Thirty-two percent of the binge drinkers indicated engaging in binge drinking one to two times per month, followed by 28% who engaged in binge drinking three to four times per month. Furthermore, 20% reported binge drinking five to six times per month and another 20% engaged in binge drinking more often than six times per month.

#### ***Binge drinking***

Because none of the adolescents was familiar with the term binge drinking, they were introduced to the definition (four or more glasses for women, five or more for men on one occasion). Almost all adolescents indicated that they did not think that this was a lot: “*Little*! *I think*! *And maybe that*’*s the average*, *but this does not do anything with us*. *Most of us wouldn*’*t even feel it*.” Their definition of a lot would rather be: drinking every day; or drinking 16 glasses of beer. Furthermore, they were asked what they would consider as drinking too much: “*You had too much alcohol when you throw up*.”, “*If you do things unconsciously*!”

#### ***Reasons not to binge drink***

Adolescents who indicated that they did not engage in binge drinking were asked what reasons they had not to do so. In four of the nine groups non-drinking adolescents could not indicate a specific reason. They just “did not do it”, or did not have any longing for alcohol “*I do not need it*. *Everybody around me drank*, *but I thought*, *no*, *I do not need that*”. One girl and a boy reported that they had very bad experiences with alcohol. The girl experienced physical impairment and black outs due to drugs in her drink. The boy once drank so much that he had to be hospitalized.

Individual sport (like cycling, boxing and swimming) at a high level also was a reason not to drink, as was having an illness that affected the liver (infectious mononucleosis). Another reason not to drink was the Islamic religion, because it forbids the consumption of any alcoholic beverage. Finally, some people just did not like the taste or the effects of alcohol or they did not expect alcohol to increase their fun when going out: “*I was on* ‘*Dancetour*’ *lately and I just drank water*. *I had the time of my life there*. *So*, *yes*, *I had as much fun* [*drinking water*] *as when I drink 10 glasses of alcohol*!” If adolescents were already classified as binge drinkers, their reasons not to drink were when they had tests at school, a sport competition, if they were sick, if they had to go to school or work the next day, or if they had to drive a car or moped.

#### ***Influence of other people on non-binge drinking behavior***

Three people mentioned bad examples from their older siblings as reasons not to drink. It was also mentioned that being in a relationship with someone prevents adolescents from binge drinking.

The answers to the question how people in their environment reacted to their non-drinking were two-fold. On the one hand, adolescents reported that almost all people in their environment liked that they did not drink, especially parents. On the other hand, many students mentioned that peers and friends offered them drinks very often. We furthermore asked if it was hard for them to resist the offering by others and again this answer was two-fold. Some of the non-drinkers said that it was no problem for them to say no, while others felt very uncomfortable and even avoided situations where alcohol was consumed: “*When they go to drink somewhere*, *I go home and sit in front of the computer*, […] *because otherwise they would offer me a drink and I would accept it*.”

#### ***Reasons to start and continue binge drinking***

Reasons for binge drinking can be classified into three categories: Drinking motives, environmental influences and alcohol expectancies. Motives to binge drink were to belong into the group when they go out on a Saturday night, to cope with negative emotions and because it is a new experience to drink alcohol. “*It is new for you if you are allowed to drink*. *Then you just try out everything*”.

Environmental cues to binge drink that were mentioned in every group were: the weekend itself, going out at the weekend, being at a party and being together with friends (at a party or at home). In some groups being a member of a sports team (e.g. soccer, hockey) was a reason to binge drink: “*On Saturday and Sunday I am usually at my hockey club*, *and yes*, *anyway*. *Uhm*…, *yes I am always drinking a couple of beers there*.” Special events like Carnival and festivals were mentioned as reasons to binge drink. Furthermore, the expectation to become more relaxed and less tense was mentioned by some people. Some adolescents indicated that the more convivial a party was, the more alcohol they drank, while on the other hand, other adolescents indicated that they expected alcohol to create a more convivial atmosphere.

#### ***Influence of other people on drinking behavior***

Adolescents stated that the size of the group affected how much they would drink. The bigger the group, the more they would consume. “*For example*, *when I am with a friend*, *we drink a couple of beers*. *But if you are with a big group*, *then you often drink a lot more*.” The opinions about whether friends and peers affect their drinking are quite different. Some adolescents said that they did not feel any pressure from peers to drink if they decided not to: “*If I made up my mind* [*not to drink alcohol*] *then there will not be much change*”. Others stated that if there is alcohol it has to be drunk or that if they are in a group, they all drink together or no one drinks. Furthermore, one person mentioned that it would matter how well you know the people you are with: “*When I am with good friends then we will drink more than when I am with people I don*’*t know very well*.”

#### ***Difficulties when trying not to binge drink***

When asked what situations made it very difficult for them not to drink or drink less than they would otherwise, most adolescents indicated that being at a party or with friends would be the most difficult situation. Most of the time adolescents reported that when they go out, they go together with friends, and they would also leave together, which makes it more difficult not to binge drink: ”*You go together with your friends into the city and you leave with them*. [*If you want to leave*] *then they say* ‘*ah*, *stay another 15 minutes*’. *But then you are there for two more hours*.” In general adolescents were convinced that the time they spent at a party significantly affected the amount of alcohol they drank. “*Yeah*, *when I come at 12 and leave at 1 a*.*m*. *I couldn*’*t drink as much as if I stayed until it was very late*, *could I*?”

#### ***Parental attitude towards alcohol consumption***

Almost all students indicated that their parents are just fine with their alcohol consumption as long as they are not so drunk that they have to throw up. “*My parents say* ‘*drinking is fine*’, *of course they would rather see me not totally drunk*, *but this happens from time to time*. *When I throw up in the house*, *then I have a problem*. *But if I am just somewhat tipsy*, *they like that*.”, “*My mom really finds it funny when I drink too much*”. Some even said that their parents would not even mind if they had to throw up, but that they had to clean it up by themselves: “*My mom always says*: *if you have to throw up*, *you clean it up*”. Some adolescents reported that there were differences between their mother and father in attitude towards drinking. “*My parents are divorced*. *He* [*the father*] *would beat me if he saw me drinking*. *I only go out when I am at my mom*’*s place*.”

#### ***Influence of parents on alcohol consumption***

Often adolescents reported that they got their first alcoholic drink from their parents. The reported ages varied between six to 15 years. Most adolescents said that the first drink was a “snow-white”, which is a mix of beer and Sprite. The first time they were binge drinking was between 13 and 15 years. Most of the adolescents reported that their first time binge drinking was outside of awareness of their parents. The occasions that were mentioned most often were: being at a party, being in a bar or during Carnival. Two times it was explicitly stated that there were older friends around the first time they were binge drinking. A few adolescents mentioned that their parents were present the first time they were binge drinking but that they were not aware of their children drinking more than 5 glasses alcohol. “*It was there* [*alcohol*] *and they* [*parents*] *did not know that I was drinking it*.”

#### ***Rules concerning adolescents’ alcohol consumption***

When asked whether their parents still handled rules concerning their alcohol consumption, almost all adolescents denied that. Some indicated that they had fixed rules before they turned 16, but that now their parents’ concern is more that they come home safely and not how much they drink or that they come home at a distinct time. “*As long as I do not have to come home alone it is always good*.” Some said that their parents would say things like: “*Take it easy*!” or “*Don*’*t drink too much*!” but never set specific limits for their alcohol consumption. Just very few mentioned that their parents had very strict ideas about their alcohol consumption and going out at night: “*I am just allowed to drink one glass of alcohol*.”

When we asked whether adolescents would respond to rules if their parents would now start to set them, reactions were divided evenly. Some said that this would have absolutely no effect on the amount they would be drinking, others said they would certainly respond to their parents rules and some were not sure (indicating that they would probably stop caring about the rules when they had been drinking 5 glasses of alcohol).

### Interview parents

#### ***Questionnaire***

In total, 18 parents participated in the focus group interviews and eight parents in one-on-one interviews (three face to face, five by telephone). Eight were male. Most parents (52%) indicated that their children had no binge drinking occasion in the previous 30 days. About 26% reported 1 or 2 binge drinking occasions in the previous 30 days and 22% stated that their children had 3 to 4 binge drinking occasions in the previous 30 days.

#### ***Attitude towards alcohol consumption***

We asked the parents about their attitude towards alcohol consumption in general of their children. Most parents indicated that they would prefer their children not to drink alcohol at all, but that they do not mind if their children drink within the limits: “*I am not against drinking*, *but it needs to be within certain limits*, *responsibly*”. Some parents were convinced that it was their child’s responsibility and that they need to trust them that they would set an appropriate limit for themselves: “*I think it*’*s useless to forbid it*. *You need to guide them to a sense of responsibility so that the child itself is able to drink within certain limits*. *It*’*s their own responsibility*. *This is what I thin*k.” We asked what the parents considered appropriate limits for their children: “*I think for a 16 year old two glasses every two to three weeks is appropriate*.” This was a limit that most parents agreed on. An advantage that parents saw in the alcohol consumption of their offspring is that they would learn to drink: “*An advantage is that they slowly learn how to drink alcohol*.” “*They can experience what it does to them*. *And I think it*’*s good if they see what it does to their peers if they drink too much*. […] *I realized from my 17 year old son that this was very impressive*. *That when others drink too much*, *that they act weird*, *experience trouble*, *become annoying*, *or situations get out of control and he did not drink at all and he became more reserved*. *I*’*m pretty sure that was influential*.”

#### ***Attitude towards binge drinking***

Just as the adolescents, parents were unfamiliar with the definition of binge drinking. We explained the meaning of the term (four standard glasses of alcohol for girls, five for boys) and then continued to ask them about their attitude towards these amounts. All parents indicated they had a negative attitude towards binge drinking. The degree of negativity varied from “*awful*”, “*dangerous*”, “*annoying*”, “*too much*” or “*not positive*”. Some parents were not happy about this behavior, but could understand that their children engage in it: “*I don*’*t think that this is something positive*, *but I can understand it a little bit*.” Some parents who already experienced binge drinking in their children tried to give an explanation for the behavior of their children: “*He is in a phase where he wants to be cool*, *cannot say no*, *and has totally no idea what this is doing to him*. *He does not know his limits*, *because he does not drink regularly*, *yet*. *If he would go out every weekend*, *then he eventually would know after four or five beers*, *that*’*s where it went wrong the last time*. *But he does not know that yet*, *and then suddenly it*’*s boom*.”

#### ***Knowledge about drinking***

Parents named a couple of physical and intellectual consequences of binge drinking. Most often parents mentioned negative consequences on brain development. Two parents specified this more: “*Look*, *if it affects your brain*, *then it affects the part involved in planning*, *organization and concentration*”. One parent in a group that mentioned effects on the brain immediately played down its negative consequences: “*On the other hand*, *when I was studying*, *I also had a period where I drank more than average*. *And I did end up quite okay*. *So I think it*’*s really hard to*… *but I think from 12 to 16 to 18 years it is*, *your brain cells are in full development*.” In second place parents mentioned liver damage and that you lose control over yourself as a consequences of binge drinking. Some parents mentioned intellectual problems as a result of binge drinking: “*Your ability to concentrate decreases*. *This affects your performance on school*.” Furthermore, a few parents mentioned having accidents, becoming comatose, having a hangover, and developing an alcohol addiction.

#### ***Parenting practices***

Almost all parents indicated that their way to reduce or control alcohol consumption in their children would be through conversation: “*The most important thing is to keep talking with your child*. *And you shouldn*’*t be blaming the child*.” Some parents mentioned that they could influence the child through their own example: “*She sees that for me it is just for social reasons*, *I like drinking a glass of wine or liquor*, *but then it stops when I have to go home by car*, *and that*’*s what she sees of course*.”

It appears that parents are quite careful when talking to their children: “*This subject should not become heavily loaded*. *Then they stop talking at all*.” “*I think you can better teach them what is the matter with alcohol rather than using rules and impose sanctions on it*.” “*Don*’*t preach to them*. *This is useless*. *And forbidding it*, *too*.”

A lot of parents indicated that they had strict rules concerning alcohol consumption before their children turned 16: “*It*’*s not allowed before you*’*re 16*. *Done*. *This was very clear for us*!” Now that their children are between 16 and 18 years, almost all parents said that they tell them that they should not drink too much, but that they do not set specific rules concerning the amount of alcohol that they allowed their children to drink. The reasons why they did not set clear limits were: “*Because you have no control about that*. *You make a fool of yourself if you tell them that they can just drink 3 glasses of alcohol*. *Do you think they comply*? *If you know their peer group and what they drink on one evening*, *you can be lucky if they have enough with 5 glasses*. *You fool yourself at the moment that you say that they cannot have more than 3 glasses*, *because you know that they do not comply*!” A lot of parents gave their lack of control as a reason not to provide strict rules concerning the amount of alcohol that they allow their children to consume. Strict rules concerning at what time they have to be home at the weekend or how long they can stay when going out were also absent. Most parents stated that they find it acceptable when their offspring come home somewhere between 1 and 2 a.m. It was also mentioned that at this age, adolescents have to learn to care for themselves: “*I think now when they slowly move towards 18 they have to become independent*. *They have to discover their own limits*, *that*’*s the best for everyone*.”

Just two parents indicated that they had absolutely no alcohol at home and that they themselves never drink alcohol. The majority of parents had alcohol available at home and the children were allowed to take it, too: “*Yes*, *we have everything at home*. *He can take a beer if he wants to*.” All parents indicated that their children were allowed to drink alcohol at home, even though they did not drink themselves or have no alcohol at home: “*They can have a glass* [*of alcohol*] *if they want to*… *but I don*’*t buy it*.” “*I think it*’*s enjoyable if the children drink a glass together with us*. *Why should they have to drink something else*?”

Finally, parents who already had experienced a situation in which their children came home drunk indicated that there were no consequences but that they had to clean up the mess by themselves: “*There was no need to talk about that*.” “*He was the idiot of the family*. *So*, *I think he has learned from that*.” Parents who had not experienced such a situation before mentioned that they would most likely have a good conversation with their child about the negative consequences of drinking. Just a few mentioned measures like restrictions on going out or pocket money.

#### ***Bringing down alcohol intake***

Parents mentioned different levels at which alcohol use in adolescents and especially binge drinking could be influenced and reduced. Firstly, many of the parents mentioned strategies that they themselves could use, like setting a good example, talking to their children about the negative consequences, or rewarding their children when they did not drink: “*We had the following agreement at home*: *If you do not smoke until you are 18*, *we will pay for your driving license*. *This kind of stimulating options*. *So I think if you make a deal with your child after they turn 16*, *like if you do not get drunk*, *then you will receive some kind of reward*.” Secondly, parents mentioned facilities like schools to engage more in education about this topic: “*The schools should present someone like an ex*-*alcoholic*, *because then they are really confronted with the negative consequences of drinking*; *then I think you can maybe reach those children*.” They also mentioned that national campaigns like the warning text on cigarette packaging should also be printed on bottles of alcohol. Also the amount and variety of alcoholic beverages should be reduced according to the parents. Special concern was also given to the Alco pops, the popular mix drinks with Rum that taste like lemonade: “*In former times* [*when we were young*] *we could choose from 4*, *5 things but now*, *the bottles get more colorful* […] *drinking alcohol within limits is okay*, *but do not bring too much on the market*. *That*’*s just inviting to try it out*.”

Finally, the current policy in the Netherlands concerning the availability of alcohol and the legal purchasing age of sixteen should change, according to some parents, in order to influence binge drinking in this age group. For example, one parent was very frustrated about the legal purchasing age of 16 years and how this undermines her authority as a parent: “*I think it is ridiculous*! *Really*, […] *when I was 16*, *there was no age limit*. *So it depended very much on your environment how they handled alcohol and what arrangements you made*. *Nowadays*, *it is legal*, *so the adolescents think*, *so*…[…] *It is regulated by law*, *so what can you do as a parent*? […] *Adolescents get an advantage here to say*: *I am 16*, *here is my ID*, *so I am allowed to drink*. *Whereas*, *if this whole regulation by law is absent then me as a parent a*: *maybe feel more responsible*, *but also b*: *make my own arrangements with my child*. *But now*, *as I just said*, *they say*, *I am 16*. […] *You have no leg to stand on*.” More parents indicated that they would like the age limit to rise to 18 years: “*The worst puberty is over with 18 years*, *and then they are more reasonable*.”

Another policy aspect that upsets some parents was the ease with which adolescents can buy alcohol in the Netherlands: “*Grocery stores shouldn*’*t sell alcohol*! […] *It is so normal*. *You buy your bread and you get your alcohol*. *No one ever thinks about that*. *I think this is highly improper of the government*.”` Many parents saw the task of bringing down binge drinking in adolescents as a responsibility of the government and catering industry: “*I consider binge drinking as terrible*. *And what especially bothers me is that this is not something of the last 5 years*, *but that it was the same 20 or 30 years ago*. *And every time they* [*the government*] *call* ‘*we are going to do something about this*’, *but the actions that are taken by the government to control this problem fail until now*. […] *This clearly is omission of the government*”. Interviewer: “*So*, *you think this is a task of the government*?” “*Yes*, *absolutely*! *This is not something of the own responsibility of people*.”

## Discussion

The current study reveals more insight into the opinion about and the handling of alcohol use within families in the Netherlands, where underage children are legally allowed to purchase and consume low strength alcoholic beverages. The unique feature of this study is that we talked to adolescents as well as parents about factors that influence binge drinking, so we could get a broad picture about what determines binge drinking in a permissive environment.

The majority of the adolescents we talked to identified themselves as a binge drinker, which is representative for the Dutch population as a whole [4]. Almost all adolescents indicated that the amount of alcohol that is defined as binge drinking (4/5 glasses of alcohol) is low. Interestingly, sports seemed to have positive as well as negative influences on drinking behavior. Non binge-drinking adolescents indicated that individual sports, like swimming or cycling, especially when at a high level, makes them refrain from drinking alcohol, whereas team sports like hockey or soccer seemed to be supportive for binge drinking events, as indicated by binge drinkers. A recent review of high school and college athletes concluded that athletes reported higher levels of alcohol consumption than did non-athletes [36]. However, there are several studies that found sports to be protective for early alcohol debut [37] and alcohol consumption [38], and has even shown to be protective against alcohol consumption when adolescents have substance using peers [39]. One study focused on sport-type differences in alcohol use among college athletes and found that swimming and diving athletes reported significantly higher levels of alcohol consumption than other sport types (Baseball/Softball, Basketball/Volleyball, Soccer, Track/Cross country) [40]. This is contrary to the results found in this study, where teams sport seems to be more encouraging alcohol consumption than individual sports. There might perhaps be a change of influence of sport teams. It could be that in late adolescence being a member of a sport team exposes adolescents more to other adolescents who are in an experimenting phase and thus encourage each other to drink together as a team, whereas when adolescents grow older and enter college a feeling of shared responsibility for the success of the team might work as an inhibitor to drink big amounts of alcohol. Another possible contributor to the higher alcohol consumption in team sports might be sponsorship of the teams by the alcohol industry. A study of New Zealand sport teams showed a positive association between alcohol industry sponsorship and AUDIT (Alcohol Use Disorder Identification Test) scores, indicating more hazardous drinking in people who are engaging in sports that are sponsored by the alcohol industry [41]. Sponsorship of sports through the alcohol industry is very common in the Netherlands (http://www.alcoholreclame.nl/alcoholreclame/alcoholreclamebeleid_in_nederland/achtergrond.html). However, we did not check in this study if the adolescents from our sample were subject to alcohol industry sponsorship.

Further, adolescents mentioned that their desire to belong to a group, and the expectation to become more relaxed were important drinking motives. This is in line with the results of a review on drinking motives [14], where social motives (to obtain social rewards) and enhancement motives (drinking to enhance a good mood or well-being) were associated with moderate and heavy drinking in young people, respectively. The review also showed that conformity motives (drinking to avoid social rejection) are hardly mentioned by adolescents; in the current study adolescents also stated to feel no peer pressure. Nevertheless, influence of friends on drinking behavior seems to be evident as it has been reported in many studies (e.g. [18,20,42]). From the literature and our study it seems that adolescents are subject to peer influences but are not explicitly aware of it. In the review of drinking motives adolescents’ ages varied between 10 and 25 years and most of the studies in this review were conducted in countries with a legal drinking age of 18 or higher. It is interesting to see that these motives seem to be the same in a more permissive society.

Environmental cues that would most likely lead to a binge drinking event, like being at a party or in a bar on weekend days together with friends, were also identified as the most difficult situations when trying not to drink. This indicates that alcohol consumption in social situations is widely accepted by Dutch adolescents. Furthermore, adolescents reported feeling pressure to drink alcohol when it is available. Further influences of peers explicitly mentioned were the size of the group and familiarity with the group. The bigger the group and the more familiar the members of the group were, the more alcohol they drank. This is in line with previous research [43]. It seems important that interventions to reduce alcohol intake in adolescents should focus on this difficult situations, strengthen their efficacy to drink in a low risk manner when much alcohol is available and provide adolescents with advice how to handle peer pressure.

Also, parental attitude towards alcohol was perceived as positive by almost all adolescents, as long as they do not get drunk and throw up. Parents themselves indicated that they regard drinking by their children to be acceptable but within appropriate limits. This confirms the adolescents’ perspective; however, those limits were defined by parents to be two glasses of alcohol every two weeks. The attitude towards binge drinking among their children was negative in almost all parents. Apparently, the views of parents and adolescents are not totally in line with each other. In an intervention to reduce alcohol use among adolescents it should be stressed that parents clearly communicate their expectations and definitions of appropriate drinking towards their children, in order to avoid misinterpretations of acceptable limits.

Research has shown that it is not only the perceived approval of alcohol consumption of peers or the approval of drinking of parents that determine alcohol consumption in adolescents but the disparity between these two [44]. The bigger the perceived gap between parental approval and peer approval, the more adolescents tended to drink. Reducing this gap may be a valuable component in an attempt to reduce alcohol consumption. Correction of the perceived norm of peer drinking [45-47] on the one hand and encouraging parents to stay involved and communicate with their children to choose friends with similar attitudes towards drinking could be possibilities to reduce the gap [44]. Interventions that encouraged parents to talk with their children about alcohol before they left for college showed that those students had less positive perceptions regarding drinking activities and showed less drinking and drunkenness [28]. Furthermore, those students also perceived their peers to have similar perceptions regarding drinking [28]. These studies indicate that parents still have considerable influence on their childrens’ drinking behavior even when they are about to leave home. When adolescents still live at home, as is the case with our target group [34], this influence should be even easier to achieve.

Further, parents were quite aware of a number of negative effects of binge drinking on the health of their children, but despite their ideas about appropriate limits and knowledge about the consequences, most parents did not set clear rules concerning alcohol use and going out. Instead, parents rather talked to their children in, an understanding, non-accusatory fashion. This again is in line with the adolescents’ reports that their parents stopped handling clear rules concerning alcohol consumption and going out when they turned 16. Parents should be encouraged to keep setting appropriate rules concerning alcohol use, as these have been proven to be effective in reducing alcohol intake among adolescents [26,27]. Also, some adolescents indicated that this would have effect on their drinking behavior, so this may be a successful strategy in at least some adolescents.

Most parents had alcohol available at home and often thought it was more safe if adolescents drank alcohol at home in their presence than outside with peers. Yet, this perception may be incorrect as one study has shown that adult-supervised settings for alcohol use, in line with harm-minimization policies are associated with higher levels of harmful alcohol consequences compared to zero-tolerance policies that favor abstinence of alcohol [48]. Often parents were convinced that their children had to learn how to drink, and that prohibiting the use of alcohol, or just allowing a certain amount of alcohol, would have no effect on the alcohol use of the child. Three factors mainly contributed to these stances: that parents experienced a lack of controllability of their child’s alcohol intake, due to the easy availability of alcohol in grocery stores; the fact that parents could not be around their children 24/7; and the legal purchasing age of 16. Some parents indicated that they had difficulties with these policies because they weaken their position as a parent. Parents explicitly stated that as soon as their children turn 16 they do not have any control about how much their child is drinking and where the child is drinking. This generally accepted, yet wrong, assumption that parents’ influence on adolescent drinking disappears when they leave home for college has been mentioned earlier [49].

Even though some parents mentioned strategies they could use to decrease the amount of alcohol their child drank, there were also a couple of parents who thought that the responsibility to reduce the problem of binge drinking lies with the legal authorities and not with themselves. This may possibly be a side effect of the permissive rule setting from the Dutch government. Hence, interventions for Dutch parents should also focus on strengthening parents’ feelings of responsibility and self-efficacy to control the alcohol intake of their child. Another possibility would be to plead to increase the legal purchasing age from 16 to 18 years. This would at least have three advantages. First, it would probably increase the feeling of control and responsibility in parents. Second, it would give a clear signal to the adolescents that underage drinking is not (no longer) acceptable and third, make it more difficult for under-age adolescents to get hold of and consume alcohol. Of course, there is a difference between the introduction of law and observing the law, as in our sample many adolescents reported drinking before the age of 16, but increasing the age limit might possibly also increase the age of first alcohol consumption. Grocery stores and bars may be more triggered to check the ID of adolescents that do not look adult and parents could also become more sensible towards providing their adolescents with alcohol when they are under 18.

The results of these focus group interviews need to be quantified using questionnaires. This would lead to more insight into which of the factors named in this qualitative research are important and changeable [50] and could give further indications on what kind of interventions to reduce binge drinking in this age group need to be developed. It is known that parents still have considerable influence on the child’s alcohol intake [25-28], and that combining parents and children in an intervention is indeed more successful than delivering separate interventions to either the child or the parent [51]. It therefore seems of upmost importance that parents are also included in these interventions to maximize the effect. Concerning the parents, we can conclude that many parents, at least from our sample, are not fully aware of the negative consequences of alcohol use, and they lack self-efficacy to control and reduce alcohol intake in their children. It also seems useful to reconsider the policies concerning the legal purchasing age and availability of alcohol to strengthen the position of parents and make availability of alcohol more difficult.

### Limitations and strengths

A limitation of focus group interviews in general is that you most likely engage with people who are motivated to talk about a certain problem. This could have particularly played a role in the interviews with parents. Parents were hard to reach and response rates were low. Despite this, we managed to get a good insight into alcohol use in Dutch families, because despite their awareness of the problem and their motivation to talk to us, these parents acted as do many parents who see no problem: i.e. not setting rules and experiencing helplessness. Furthermore, we had to use one-on-one interviews with some parents, because for them it was not possible to join a focus group (e.g., because of the distance or time constraints). A disadvantage of this method is that you miss discussion with other parents in the group, but a big advantage is that you can get more in-depth insights in comparison with focus group interviews. Finally, we relied on self-reports of adolescents and parents, which can be prone to subjective bias, and due to a lack of insight information from self-report data can be missing.

The major strength of this study is that we combined focus group interviews from adolescents and parents, which creates a broader view on the problem and possible solutions to reduce binge drinking in adolescents.

## Conclusions

Dutch parents and adolescents are facing a unique problem. Even though adolescents aged 16 are not grown up, they are allowed to buy low strength alcoholic beverages and are expected to engage in low risk drinking. In reality this often does not take place, as excessive consumption of alcohol is a big problem in the Netherlands. We gained insight into the reasons why Dutch adolescents binge drink and how Dutch families handle alcohol consumption. We come to the conclusion that there are many opportunities to intervene, in particular through combined parent–child interventions, in order to improve the way families deal with alcohol and ultimately reduce the alcohol intake in this age group.

### Consent

Oral informed consent was obtained from every participant for the use of these data for scientific research and publication.

## Competing interests

The authors declare that they have no competing interests.

## Authors’ contributions

AJ contributed to the development of the semi-structured questionnaire; conducting the interviews, transcribing the audiotapes; coding the transcripts; analysing and interpreting the data; and writing the manuscript. RC contributed to the development of the semi-structured questionnaire; conducting the interviews; coding the transcripts; interpreting the data; and drafting and revising the paper. LM contributed to the development of the semi-structured interview; interpreting the data; and drafting and revising the paper. HdV contributed to the developed the semi-structured questionnaire; interpreting the data; and revising the paper. All authors read and approved the final manuscript.

## Pre-publication history

The pre-publication history for this paper can be accessed here:

http://www.biomedcentral.com/1471-2458/13/882/prepub
